# Unequal benefits: the effects of health insurance integration on consumption inequality in rural China

**DOI:** 10.3389/fpubh.2025.1490393

**Published:** 2025-05-02

**Authors:** Linlin Han

**Affiliations:** ^1^School of Economics and Management, Qilu Normal University, Jinan, Shandong, China; ^2^School of Economics, Shandong Normal University, Jinan, Shandong, China

**Keywords:** health insurance, urban–rural health insurance integration, consumption inequality, rural residents, relative deprivation index, staggered difference-in-differences method

## Abstract

**Introduction:**

Unlocking the consumption potential of rural residents and narrowing the consumption gap is crucial for expanding domestic demand and enhancing social equity. This study examined how integrating Urban-Rural Residents Medical Insurance (URRMI) affected consumption inequality among rural residents and its underlying mechanisms.

**Methods:**

We analyzed 17,092 observations from the China Family Panel Studies (CFPS) 2012-2018. Consumption inequality was measured using the Kakwani relative deprivation index. A staggered difference-in-differences (DID) design with high-dimensional fixed effects was employed to analyze the impact of the urban-rural health insurance integration policy on consumption inequality. Robustness checks such as placebo tests, heterogeneity in treatment effects, and spatial spillover analyses were addressed.

**Results:**

The findings reveal that the policy significantly raises consumption levels among middle and high-income groups while concurrently reducing expenditures for the lowest-income bracket, exacerbating consumption inequality. Heterogeneity analysis indicates that the impact of urban-rural health insurance integration on rural consumption inequality is manifested in both consumption structure and life-cycle effects, with the most significant disparities observed in subsistence and enjoyment consumption, particularly among middle-aged and older age groups. Mechanism analysis identifies increased utilization of medical services, the release of precautionary savings among middle and high-income cohorts, and variations in health insurance funding modalities as key drivers of the widening consumption inequality gap.

**Discussion:**

The study concludes with recommendations to promote the establishment of parity in urban-rural integrated health insurance and to prioritize policy support for vulnerable groups, especially the older adult and impoverished households.

## 1 Introduction

As China has become the largest developing country and its economy has achieved significant milestones, the aspiration for common prosperity amidst high-quality development has emerged. However, the country currently grapples with pronounced inequalities (1). The persistent expansion of disparities in resident income, wealth, and consumption, alongside the limited redistributive role of the social security system, has been a longstanding concern (2). These inequalities are evident not only in the differential access to welfare and public services across regions and between urban and rural areas but also in the social stratification within the same group regarding individual living welfare conditions (3). Consumption, a vital component of welfare, provides a comprehensive reflection of individual living standards. Specifically, consumption inequality can directly and vividly reveal the living welfare status of individuals (4). Compared to the higher-income groups with better health, the lower-income groups with poorer health face a distinct disadvantage in consumption expenditure (5). This individual-level consumption gap continues to damage the health capital of disadvantaged groups through the relative deprivation of their physiological health and the perception of psychological differences, leading to a range of diseases and long-term deprivation of their labor capacity. This exacerbates the existing consumption inequality and perpetuates it across generations (6). This situation further highlights the issue of wealth disparity and necessitates a focus on enhancing the consumption level of lower-income groups to effectively alleviate the consumption gap between different income levels, thereby mitigating social conflicts.

Equity and fairness are primary policy objectives in the healthcare domain (7). Despite being established since the 1990s, China’s medical insurance systems, including the Urban Employee Basic Medical Insurance, the New Rural Cooperative Medical Scheme for rural residents, and the Urban Residents Basic Medical Insurance, have had limited success in enhancing healthcare equity. In recent years, the Chinese government has pursued the integration of the New Rural Cooperative Medical Scheme with the Urban Residents Basic Medical Insurance into a singular framework known as the Urban–Rural Residents Medical Insurance (URMI), aiming to expand health insurance coverage and enhance the accessibility and affordability of medical services (8). According to the National Medical Security Development Statistics Bulletin, in the year following the implementation of the policy, the coverage of the URMI expanded rapidly to 875 million individuals, accounting for approximately 62.5% of the national population. By the end of 2023, the number of URMI participants had reached 983 million, representing 69.84% of the total population, making it the largest and most extensive medical insurance scheme in China. The primary aim of this integration is to enhance the medical insurance entitlements for non-employed individuals in urban and rural areas, thereby promoting parity in medical insurance coverage between these two demographic groups (9, 10). In accordance with the policy objectives, the integration of health insurance should ideally lead to the dispersion of health risks among groups with varying levels of health and financial status. This is to be achieved through the refinement of income redistribution mechanisms, with the ultimate aim of reducing the socioeconomic disparities that can arise due to health-related issues. Nevertheless, the health insurance system in China faces challenges due to limited economic capacity to provide a high level of equitable coverage. Consequently, the phenomenon of “reverse compensation,” where middle- and high-income groups disproportionately benefit from health services utilization compared to low-income groups, persists and remains a significant issue (9, 10). However, previous studies have not yet assessed the policy effects of urban–rural health insurance integration from the perspective of consumption inequality. In addition, during the process of integrating urban and rural health insurance, some regions have experienced issues such as delayed policy rollout, rapid increases in individual payment standards, and significant differences in existing systems, which hinder the income redistribution regulatory role of the integrated URMI (11). In light of this, this paper attempts to theoretically analyze and empirically test potential problems in the process of URMI integration reform and its impact on consumption inequality and underlying mechanisms, providing useful references for the improvement of the design of the health insurance system.

Based on the perspective of consumption inequality, this paper explores the policy effectiveness of urban and rural health insurance integration in rural areas. Initially, the characteristic fact of consumption relative deprivation within rural households is measured using the Kakwani Relative Deprivation Index, based on data from four phases of the CFPS. Subsequently, the impact of urban and rural health insurance coordination on consumption inequality is empirically examined using the staggered DID model, with heterogeneity analysis across consumption structures and life-cycle dimensions. Finally, this paper assesses the mechanisms that may exacerbate consumption inequality in rural areas, including the utilization of health services, precautionary savings, and health insurance financing models.

The potential contributions of this study are as follows: First, it pioneers the evaluation of benefit equity within the healthcare system from a consumption perspective. Integrating consumption relative deprivation into the analytical framework leads the way in healthcare benefit equity, which traditionally focuses more on health-related inequalities. Second, it broadens the scope of consumption inequality research. While existing literature predominantly examines consumption gaps between urban and rural areas using macro indicators, this paper constructs relative deprivation indicators to assess consumption disparities at the individual level, providing a nuanced examination of consumption differences within rural areas. Third, it uses a precise method of causal identification. Considering the exogenous and progressive implementation characteristics of the urban and rural health insurance coordination reform, the article constructs a staggered DID method with high dimensional fixed effect. Furthermore, this paper uses multiple scientific robustness tests to verify the causal relationship between urban and rural health insurance coordination and consumption relative deprivation.

## 2 Background and literature review

### 2.1 Background

China has historically confronted significant inequalities in health, medical service utilization, and welfare among different populations due to its three primary health insurance systems segmented by urban–rural divide, regions, and population groups (12). The New Rural Cooperative Medical Scheme, established for rural non-working individuals, has long provided a lower level of medical security compared to the other two insurance systems, aiming to mitigate future uncertainties and bolster consumption decisions for rural households (13). In response to these disparities, provinces such as Qinghai, Chongqing, Tianjin, Ningxia, and Guangdong took the lead in integrating the Urban Residents Medical insurance and the New Rural Cooperative Medical Scheme into a unified system after 2008, attempting to ensure that urban and rural non-working populations receive equivalent medical protection. In January 2016, the State Council of China issued the “Opinions on the Integration of Basic Medical Insurance Systems for Urban and Rural Residents,” outlining the fundamental principles and specific requirements for the integration of health insurance for urban and rural residents, resulting in the establishment of the unified URMI. The expanded health insurance adheres to the basic requirements of higher reimbursement rates, a more comprehensive health insurance catalog, and an increased number of designated medical facilities, demonstrating significant enhancements in reimbursement rates, designated medical institutions, pharmaceutical catalogs, and coverage for major illnesses, thereby surpassing previous standards (14). The integration of the URMI has played a pivotal role in narrowing the disparity in health insurance benefits between urban and rural dwellers, thereby establishing a robust framework to mitigate health risks, lighten the financial strain associated with medical treatment, and stimulate consumption choices. Due to the implementation of this health insurance reform in China, the most populous developing country in the world, it has attracted widespread attention from domestic and international scholars.

From a financial perspective, the funding modalities for integrated URMI across regions can be broadly classified into two categories: a single-tier payment system and a multi-tier payment system (58). The single-tier system requires uniform premiums and benefits for all insured individuals, whereas the multi-tier system permits adult enrollees to select from multiple premium levels, with benefits directly proportional to the contributions made. The majority of cities have established two premium levels based on the previous standards of Urban Residents Medical Insurance and the New Rural Cooperative Medical Scheme. A minority of regions have introduced three-tier or more complex systems, which either include an intermediate tier or cater to families with serious illnesses or higher economic status, aligning with the principles of the tiered system. The effectiveness of this policy in achieving equity has been a focal point of interest for both the state and the populace. However, given the recent completion of the integration process, research into the policy’s impact on equity remains in its infancy and requires further in-depth exploration.

### 2.2 Literature review

The trends and measurement of consumption inequality. Regarding the trends of consumption inequality, studies have found that the level of consumption inequality in various countries has generally increased over the past half-century. For instance, in the context of the United States, Attanasio and Pistaferri (15) noted that consumption inequality in the U.S. remained stable in the 1970s and grew rapidly after the 1980s. In contrast, Krueger and Perri (16) argued that there was no significant increase in consumption inequality in parallel with income inequality in the U.S. between 1972 and 1998. They attributed this to the rapid development of the domestic credit market during the same period, which provided households with means to smooth consumption. Aghapour et al. (17) indicated that the consumption inequality for out-of-pocket medical expenses in Iran slightly decreased between 1984 and 2019, and this inequality is higher among affluent families, such as those with insurance coverage and those in higher-income brackets. Cai et al. (18) and Li et al. (14) explored consumption inequality trends in China, revealing a pattern of initial rise followed by a decline. Measurement methods for consumption inequality can be categorized into group-level and individual-level indicators. Common group indicators include the Gini coefficient (19), the Atkinson index (20), the generalized entropy index (21), and the interquartile ratio (22), which provide insights into societal or group-level consumption disparities. However, recent studies have increasingly focused on individual-level measurements to address consumers’ specific concerns about the relative deprivation they face. Individual-level indicators like the Yitzhaki index, the Podder index, and notably, the Kakwani index have gained prominence due to their dimensionless, regularized properties, with the mean being the Gini coefficient (6, 23, 24). The Kakwani index calculates the consumption gap between an individual and all others in their reference group with higher consumption levels, offering insights into inequality induced by upward social comparisons within reference groups.

The causes of consumption inequality. From the perspective of household heterogeneity characteristics, income inequality emerged as a pivotal determinant of consumption inequality (16). The reduction in consumption inequality among young people is partly attributed to the increase in property values, which has led to both the wealth effect and the so-called “mortgage slave” effect (25). The improvement of financial literacy has reduced the sense of relative deprivation among rural households by facilitating credit smoothing, asset appreciation, and insurance protection. For instance, rural residents with higher financial literacy are more likely to use loans for investment or production expansion, thereby increasing their income and consumption levels (26). From the perspective of the external environment, the presence of state-owned enterprises may reduce consumption inequality by providing stable employment and income, while issues in the urbanization process, such as the widening urban–rural divide and the imperfect social security system, may exacerbate consumption inequality (18, 27). For instance, globalization has brought trade liberalization that offers consumers cheaper goods and services, raises income levels, and effectively curbs consumption inequality (28). Furthermore, interventions in digital finance, such as the widespread adoption of mobile payments and online banking services, have reduced consumption inequality by lowering the cost of financial services and enhancing financial inclusion (29). Government policies also played a crucial role in shaping consumption inequality. Chu (30) developed a quality-ladder growth model with wealth heterogeneity and elastic labor supply, finding that the impact of strengthened patent protection on consumption inequality depends on the elasticity of intertemporal substitution, and while it increases income inequality, its effect on consumption inequality is relatively small based on U.S. data calibration. Komatsu (31) utilized a Search-and-Matching TANK model with sticky wages to analyze the impact of monetary policy on consumption inequality, finding that an expansionary monetary policy decreases inequality by operating through the income composition channel, the savings redistribution channel, and the earnings heterogeneity channel, with the latter playing a significant role in models that incorporate wage rigidities. Progressive consumption tax rates and reforms in personal income tax systems have been recognized as effective tools in reducing wealth and consumption inequality. According to Khieu and Van Nguyen (32), by designing tax structures in a way that imposes higher tax rates on higher levels of income and consumption, governments can redistribute wealth more equitably. In addition to taxation policies, pro-poor transfers have demonstrated potential in mitigating urban–rural consumption inequality by directly boosting the purchasing power of low-income households, as indicated by Aaberge et al. (33). Research on pension insurance revealed that income growth and income gap mitigation effects are mechanisms by which pension insurance reduces inter-individual relative deprivation, but the coexistence of multiple pension insurances also increased household consumption inequality (34).

Economic impact of health insurance. Studies have consistently shown that health insurance can significantly influence financial well-being and consumption patterns. For example, Finkelstein et al. (35) conducted a seminal study on the Oregon healthcare experiment, revealing that expanding health insurance coverage led to a substantial increase in healthcare utilization and a notable reduction in financial strain among low-income individuals. The work of Cai et al. (18) has shed light on the role of health insurance in promoting household durable goods consumption in China, with urban households buying refrigerators, washing machines, and air conditioners, and rural households buying color TVs, refrigerators, washing machines, air conditioners, and computers. Kolukuluri (36) articulated that health insurance plays a role akin to partial insurance within the realm of household expenditure patterns. The study suggests that the availability of health insurance is instrumental in dampening the volatility of consumption expenditures on food and healthcare services during periods of health-related adversity faced by families. Nonetheless, it is observed that the protective capacity of health insurance against the downturn in non-food consumption is not fully robust. From the perspective of expanding health insurance coverage, a study found the expansion of Medicaid coverage under the Affordable Care Act significantly increased the utilization of preventive health services and overall health status (37). Hu et al. (38) conducted a study examining the impact of the Affordable Care Act (ACA) on the financial well-being of the American population, revealing that the expansion of Medicaid significantly improved the financial status of low-income adults. Specifically, this policy led to a reduction in the number of unpaid bills and the amount of debt referred to third-party collection agencies among individuals residing in states that expanded Medicaid, who were of lower income and previously uninsured. Saksena et al. (39), based on the experience in Rwanda, demonstrated that the expansion of health insurance is associated with a higher degree of financial risk protection. Recent research has intensively examined the impact of China’s urban–rural health insurance integration policy on household consumption and economic burden. Huo et al. (40) demonstrated that the policy of integrating urban and rural health insurance has played a pivotal role in reducing the financial strains associated with healthcare, particularly among the older adult, rural populations, and those engaged in urban–rural migration. Chen et al. (41) indicated that the integration policy has led to an average increase of 14% in non-medical consumption for the treatment group. This effect is particularly pronounced in non-food consumption, as the price elasticity of demand for non-food items is greater than that for food items. Wang and Hu (42) discovered a robust positive correlation between the integration of health insurance systems and the escalation of consumption levels, encompassing both essential subsistence expenditures and overall household consumption, within the demographic of rural households led by middle-aged and older adults. Specifically, the unified urban–rural health insurance framework achieves this by alleviating the economic strain imposed by medical costs, which in turn augments health indices and extends life expectancy. Collectively, these studies indicate that the policy has not only elevated the level of medical protection for rural residents but also increased consumption of non-food and essential items by reducing household medical expenditures and precautionary savings, thereby enhancing their overall welfare. The existing body of literature has not extensively explored the effects of health insurance on household consumption inequality. Burkhauser and Simon (43) posited that health insurance reform, by expanding coverage and offering subsidies, can mitigate economic inequality, particularly exerting a significant positive impact on low-income households. Johar et al. (44) found that Indonesians’ access to health care was pro-rich in general but pro-poor at Puskesmas. Zhou and Huang (45) compared the effectiveness of China’s basic health insurance in alleviating the relative deprivation of rural migrant workers and found that there are large differences in the fairness of the benefits of different health insurance policies. Unfortunately, their studies did not examine the effect of URMI. Moreover, there is a gap in research concerning the influence of the integration between urban and rural health insurance systems on consumption inequality, which warrants further investigation.

The underlying mechanisms. Health service utilization and healthcare burden, precautionary savings, and insurance funding modalities may be the mechanisms through which URMI affects household consumption inequality (18, 35, 42, 43). From the perspective of healthcare service utilization, Finkelstein et al. (35) demonstrated that the Oregon healthcare reform program notably increased healthcare service utilization among low- and middle-income groups in the United States. However, Wagstaff et al. (46) and Serrano-Lomelin et al. (47) observed prevalent healthcare service utilization inequality resulting from health insurance expansion. Tangcharoensathien et al. (48) noted that Thailand’s Universal Coverage Scheme (UCS) promotes equity in health financing and enhances the accessibility and affordability of medical services in Thai society by providing a comprehensive health benefits package and ensuring financial risk protection, demonstrating a pro-poor nature. With integrated urban–rural health insurance, low-income groups may augment medical service utilization, potentially reducing instances of foregoing treatment (49). Yet, this could inadvertently escalate total medical costs. If the risk compensation mechanism fails to offset rising medical expenses, low-income groups may see a surge in out-of-pocket medical spending (50). Furthermore, urban–rural health insurance integration might exacerbate disparities in medical service utilization, as high-income groups with better health conditions potentially enjoy more comprehensive medical services, while the medical burden on low-income groups with poorer health conditions escalates (10). Examining the role of precautionary savings, precautionary savings refer to the accumulation of savings by households to cope with the uncertainty of future income and potential risks (51). In the context of Constant Relative Risk Aversion (CRRA) preferences, where households exhibit constant attitudes toward risk regardless of their wealth level, families with higher initial endowments may hold more precautionary savings due to the uncertainty of future asset returns (52). Compared to the previous “New Rural Cooperative Plan,” the URMI offers superior medical security benefits, which helps to alleviate residents’ concerns about future health expenditures, thereby unleashing their consumption potential. This could lead to higher-income groups allocating more of their precautionary savings toward consumption, potentially widening the degree of consumption inequality among different households (53). However, expenditures on health insurance premiums exert an erosive effect on assets and reduce household disposable income, which disproportionately affects the budget constraints of low-income families and thereby exacerbates consumption inequality (54). Lastly, the funding modalities transition is a critical aspect of health insurance integration. An important objective of the integration of urban and rural health insurance is to achieve uniform premium payments and equal benefits for all insured individuals. However, during the reform process, some regions have implemented a single-tier system where all insured individuals pay the same premium, while others have adopted a multi-tier system with different premium amounts corresponding to varying levels of medical coverage (58). This multi-tiered model primarily employs a gradual transition approach, which will eventually convert to a single-tier system as household income levels generally increase (11). In a single-tier system, the limited payment capacity of low-income groups may lead to unbearable financial pressure due to a substantial short-term increase in premiums, potentially suppressing the consumption potential of this demographic directly. In contrast, a multi-tier system, by fully considering the payment capacity of insured families, can prevent the short-term financial strain on middle and low-income groups, which may contribute to reducing consumption inequality.

## 3 Data variables and models

### 3.1 Sample data

This study utilizes the China Family Panel Studies (CFPS) database from 2012, 2014, 2016, and 2018 as its research sample. CFPS, administered by Peking University, constitutes a comprehensive and nationwide longitudinal survey, providing a rich dataset for analysis on a wide array of social phenomena in China (59). The initial survey, conducted in 2010, comprised 14,960 households and 42,590 individuals across 25 provinces in China, with follow-up surveys occurring biennially. The CFPS collects a wide range of information, including economic activity, insurance participation, family dynamics, population migration, and physical and mental health, offering a robust foundation for our research. The data processing procedure is as follows: First, we designate the “financial respondent” as the household head’s proxy and align household characteristics accordingly. Subsequently, we identify households that participated consistently across all four survey periods, ensuring data balance. Observations with urban domicile, health insurance enrolment status of not enrolled, missing control variables, and anomalies are excluded. Further refinement involves removing the highest and lowest 1 percent consumption levels samples and retaining households headed by individuals aged between 16 and 80 years. Following these procedures, our dataset comprises 17,092 observations from 4,273 households, providing a robust foundation for our analysis.

### 3.2 Variables

#### 3.2.1 Dependent variable

Household consumption inequality, denoted as *RD* (Relative Deprivation), is assessed using the Kakwani relative deprivation index, as outlined by Zhou and Huang (45). Given the organizational framework of the current urban and rural health insurance system, which operates at the city or district level, households tend to select comparison samples from nearby geographical areas with higher consumption levels. Consequently, individuals residing in the same district as the surveyed household are chosen as the reference group for comparison. The consumption inequality index is then derived by contrasting the surveyed household with others in the district with higher consumption levels.

The calculation methodology is delineated as follows: Let *X* represent the reference group, with *N* denoting the total number of households within the group. These households are ranked based on ascending consumption levels, resulting in a consumption vector distribution represented as *C = (c_1_, c_2_, c_3_*,…, *c_N_)*, where *c_1_ ≤ c_2_ ≤ c_3_ ≤ … ≤ c_N_*. Here, *c_i_* represents the consumption level of the ith household within the group. By comparing the current consumption level of household *i* with that of other household *j* within the same cluster, the relative inequality can be expressed as:


(1) $$RD\left(c_{i},c_{j}\right)=\left\{\begin{array}{l} c_{j}-c_{i},for\;c_{j}>c_{i}\\ 0\;,for\;c_{j}\leq c_{i} \end{array}\right.$$

In Equation 1, the indices *i* and *j* are constrained to satisfy 1 ≤ *i*, *j* ≤ *N*. This formulation signifies that a relative deprivation exists when the consumption level of household *j* exceeds that of household *i* (*c_j_* > *c_i_*). Conversely, no relative deprivation occurs when the consumption level of household j is equals or lower than that of household *i* (*c_j_* ≤ *c_i_*).

Let us define some terms: *u_ci_^+^* represents the mean consumption across all households within the cohort that surpasses *c_i_*, *N_ci_^+^* signifies the total number of households consuming more than *c_i_*, and*γ_ci_^+^* represents the proportion of such households within the total sample. The Kakwani consumption relative deprivation index for household *i* is computed by summing *RD(c_i,_c_j_)* over all households *j* and dividing by the mean consumption of households within the cluster and the total sample size:


(2) $$\begin{array}{l} RD\left(c_{i}\right)=\frac{1}{Nu_{x}}\overset{N}{\underset{j=1}{\sum }}RD\left(c_{i},c_{j}\right)=\frac{1}{Nu_{x}}\left(N_{c_{i}}^{+}\times {u_{c_{i}}}^{+}-N_{c_{i}}^{+}\times c_{i}\right)\\ \;=\frac{1}{u_{x}}{\gamma_{c_{i}}}^{+}\left({u_{c_{i}}}^{+}-c_{i}\right) \end{array}$$

In Equation 2, *RD*(*c_i_*) quantifies the relative deprivation experienced by household *i* concerning all other individuals within the same cohort who consume at a higher level than household *i*. This metric, bounded between 0 and 1, serves as a monotonically decreasing function of household consumption.

#### 3.2.2 Independent variable

The implementation of the urban–rural health insurance coordination policy (insurance) serves as an exogenously driven government policy reform. This reform acts as a quasi-natural experiment, facilitating a comparative analysis of consumption inequality changes in rural areas pre- and post-policy implementation through a difference-in-differences model. The urban–rural health insurance integration policy is rolled out gradually across different regions. The determination of whether this policy is implemented in a specific region and the timing of implementation are established by gathering coordination implementation plans issued by respective government departments in each region, along with their issuance dates. The variable insurance_ijt_ assigned a value of 1 for the current year and subsequent years if the policy has been enacted in the city where the household resides; otherwise, it is assigned a value of 0.

#### 3.2.3 Control variables

Drawing upon the research of Pak (24) and Li and Zhang (34), this paper selects control variables from three primary levels. Firstly, demographic characteristics encompass the gender of the household head (with males coded as 1 and females as 0), age and its square to capture non-linear effects on consumption inequality, marital status (1 for married and 0 otherwise), educational attainment (classified, with respective values assigned from 0 to 7), employment status (1 for those employed and 0 for those unemployed or not in the labor force), working hours (represented by the number of hours worked per week), and self-assessed health status (classified, 1 to 5 based on the household head’s evaluation of their health condition). Secondly, household characteristics encompass indicators of income (logarithm of household income plus 1), assets (logarithm of financial assets and real estate value plus 1), population size, participation in pension and health insurance schemes, proportion of population in the educational stage, proportion of population over 65 years old, and self-assessed socio-economic status (classified, 1 to 5 based on the household’s evaluation of their socio-economic condition). Lastly, regional characteristics, encompassing the per capita GDP of each city, the per capita health expenditure of each province, and the geographical location of each city (whether in Eastern, Central, or Western China).

### 3.3 Descriptive statistics

Descriptive statistics for each variable are presented in Table 1. According to the descriptive statistical results of the outcome variables, the treatment group exhibits higher levels of total consumption inequality, as well as inequality indices for subsistence, development, and enjoyment consumption, compared to the control group. It is suggested that the reform of the urban–rural health insurance integration policy may significantly contribute to the increased consumption inequality among rural residents. This hypothesis will be further tested and validated through subsequent empirical research. The descriptive statistics for the control variables reveal no significant disparities between the treatment and control groups with respect to several dimensions: age, marital status, educational attainment, employment status, working hours, self-assessed health, engagement in medical and pension insurance schemes, self-rated socio-economic status, and regional attributes, throughout the sampling period. This absence of significant variation implies a commendable level of balance within the study’s sampled data. Nevertheless, a detailed examination of the baseline year data, specifically from 2012, discerns a higher prevalence of male household heads, reduced household income and financial assets, and a greater number of family members within the group subjected to the urban–rural health insurance integration. These observations indicate subtle yet pertinent distinctions in household head gender, asset possession, and family size between the treatment and control groups, which may complicate the mitigation of endogeneity in this analysis. To address this, the subsequent section on robustness testing incorporates these initial differences, along with their temporal trajectories, into the regression models, thereby enhancing the study’s methodological rigor and the reliability of its findings.

**Table 1:** Descriptive statistics.

Variable	2012		2014		2016		2018	
	*t* = 0	*t* = 1	*t* = 0	*t* = 1	*t* = 0	*t* = 1	*t* = 0	*t* = 1
*RD*	0.402	0.433	0.413	0.501	0.422	0.465	0.379	0.455
*RDLIV*	0.399	0.429	0.415	0.486	0.418	0.545	0.343	0.424
*RDDEV*	0.559	0.568	0.518	0.481	0.532	0.541	0.536	0.550
*RDENJ*	0.857	0.857	0.704	0.806	0.695	0.709	0.702	0.767
*gender*	0.423	0.586	0.522	0.544	0.514	0.536	0.517	0.586
*age*	48.10	48.31	50.61	51.43	51.46	51.97	52.80	53.31
*age2*	24.89	25.24	27.31	28.24	28.38	28.40	29.88	30.18
*marr*	0.797	0.741	0.884	0.868	0.864	0.898	0.886	0.910
*edu*	1.525	1.445	1.765	1.886	1.563	1.268	2.027	1.787
*work*	0.621	0.622	0.619	0.621	0.622	0.620	0.610	0.609
*hours*	47.113	46.590	45.937	47.433	48.527	46.323	47.047	48.235
*health*	3.466	3.471	3.660	3.572	3.319	3.407	3.006	3.135
*income*	10.06	9.84	10.27	10.23	10.30	10.36	10.44	10.13
*finance*	0.375	0.325	0.512	0.505	0.584	0.423	0.647	0.614
*house*	1.314	1.225	2.355	2.224	2.487	2.394	3.003	2.478
*famnu*	3.995	4.224	3.857	3.798	3.793	3.975	3.607	3.884
*penins*	0.175	0.152	0.484	0.491	0.505	0.477	0.519	0.531
*medins*	0.899	0.879	0.927	0.904	0.933	0.905	0.937	0.955
*Proportion65*	0.311	0.322	0.334	0.351	0.320	0.319	0.287	0.293
*Proportionedu*	0.320	0.329	0.387	0.363	0.317	0.307	0.393	0.371
*status*	3.088	3.045	3.160	3.171	3.035	3.101	3.067	3.048
*perGDP*	10.598	10.590	10.751	10.756	10.831	10.892	11.012	11.019
*perEXP*	7.244	7.235	7.481	7.472	7.902	7.910	8.031	8.049
*area*	1.843	1.833	1.841	1.832	1.872	1.890	1.835	1.841

*t* = 1 indicates the treatment group that implemented the urban–rural health insurance integration, and *t* = 0 indicates the control group that did not participate in the integration. *, **, and *** indicate the 10%, 5%, and 1% statistical significance levels.

### 3.4 Primary analysis of consumption inequality

**Figure 1:**
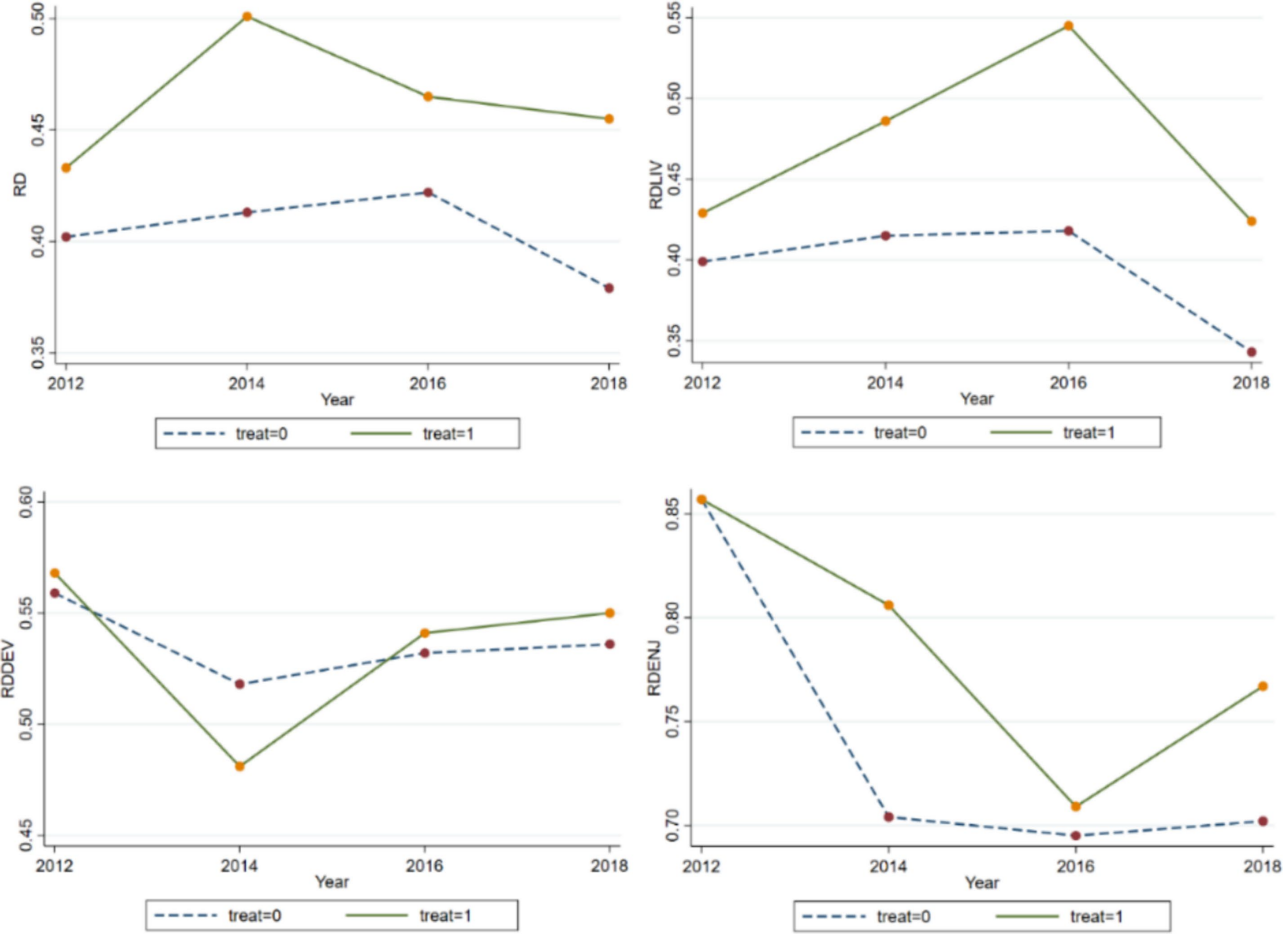
Overall and sub-consumption inequality indices. RD, RDLIV, RDDEV, and RDENJ represent the total consumption inequality index, the subsistence consumption inequality index, the development consumption inequality index, and the enjoyment consumption inequality index, respectively.

Figure 1 presents an analysis of consumption relative deprivation trends in rural areas using data from CFPS from 2012 to 2018. Numerical examination reveals that household consumption inequality has remained within the range of 0.402 to 0.501, while the subsistence consumption inequality index, ranging from 0.399 to 0.545, closely mirrors the overall consumption inequality level. Notably, enjoyment consumption exhibits the highest level of inequality among various consumption types. A closer examination of the trend indicates an inverted U-shape distribution for both overall and subsistence consumption inequality indices, with peaks observed in 2014 and 2016, respectively, followed by gradual declines. This suggests a narrowing gap in consumption levels and subsistence expenditures among rural households in China in recent years. Conversely, development and enjoyment consumption inequality indices display a positive U-shaped trend, indicating a gradual expansion of such expenditures among rural households. Furthermore, households participating in the urban–rural health insurance coordination exhibit notably higher consumption relative deprivation compared to non-participating households, except for relative deprivation in development consumption.

### 3.5 Empirical models

Given that the urban–rural health insurance integration policy constitutes an exogenous shock for households, prior research has commonly employed the difference-in-differences (DID) method to estimate changes in household health or healthcare burden (50, 53). However, owing to the policy’s gradual rollout across different regions, a staggered DID approach, as suggested by Beck et al. (55), avoids explicitly defining a control group. Instead, it designates a control status based on whether urban–rural health insurance integration is implemented in the district of each household before policy implementation. Subsequently, this control status transitions to an treatment group post-implementation. The model setup is delineated as follows:


(3) $$RD_{ijt}=\beta_{0}+\beta_{1}\mathrm{insuranc}e_{ijt}+\beta_{2}X_{ijt}+\lambda_{j}+u_{t}+\lambda_{j}\times u_{t}+\varepsilon_{ijt}$$

*RD_ijt_* denotes the degree of relative inequality in consumption (Kakwani relative deprivation index) in period *t* for the *ith* household located in district *j*; *insurance_ijt_* denotes whether urban–rural health insurance integration has been implemented in period *t* in district *j* where household *i* is located and is assigned the value of 1 after the implementation of the policy, and 0 otherwise; and *X_ijt_* is the head, household, and region characteristics variables. *λ_j_* and *μ_t_* are the city and time fixed effects, and the interaction terms of the city and the time fixed effects are also added to further rule out the effect of heterogeneous trends in consumption inequality across different areas. The coefficient *β_1_* measures the extent to which the implementation of the urban–rural health insurance integration policy in rural areas affects relative consumption inequality.

## 4 Empirical result analysis

### 4.1 Baseline regression results

Table 2 presents the regression outcomes examining the impact of urban–rural health insurance integration policy implementation on household consumption relative deprivation. Columns (1) to (4) progressively incorporate control for area and time fixed effects, along with head, household and region characteristics. The results across the four columns consistently indicate a significantly positive effect of urban–rural health insurance integration implementation in rural areas on the Kakwani index at the 1% significance level. This finding suggests that the policy exacerbates consumption inequality among rural households.

**Table 2:** Benchmark regressions results.

Variable	(1)	(2)	(3)	(4)
*insurance*	0.071*** (0.015)	0.058*** (0.014)	0.040*** (0.013)	0.032*** (0.011)
*gender*		0.004* (0.002)	0.006*** (0.002)	0.007*** (0.002)
*age*		−0.003*** (0.001)	−0.000 (0.001)	−0.003*** (0.001)
*age2*		0.008*** (0.001)	0.004*** (0.001)	0.003*** (0.001)
*marr*		−0.103*** (0.004)	−0.045*** (0.009)	−0.035*** (0.009)
*edu*		−0.027*** (0.011)	−0.018*** (0.007)	−0.014*** (0.005)
*work*		−0.011** (0.005)	−0.009** (0.004)	−0.007** (0.003)
*hours*		−0.005 (0.004)	−0.004 (0.003)	−0.002 (0.002)
*health*		−0.020*** (0.007)	−0.018*** (0.006)	−0.012*** (0.004)
*income*			−0.040*** (0.012)	−0.030*** (0.011)
*finance*			−0.009*** (0.003)	−0.008*** (0.003)
*house*			−0.028*** (0.010)	−0.025*** (0.010)
*famnu*			−0.033*** (0.010)	−0.030*** (0.010)
*penins*			0.005* (0.003)	0.004* (0.002)
*medins*			−0.002 (0.004)	0.002 (0.002)
*Proportion65*			0.010*** (0.03)	0.008*** (0.02)
*Proportionedu*			−0.009*** (0.003)	−0.007*** (0.002)
*status*			−0.012** (0.006)	−0.010** (0.004)
*perGDP*				−0.002* (0.001)
*perEXP*				−0.035*** (0.009)
*area*				0.014** (0.007)
constant	0.404*** (0.001)	0.471*** (0.015)	0.594*** (0.016)	0.517*** (0.020)
area FE	YES	YES	YES	YES
time FE	YES	YES	YES	YES
Obs	17,092	16,948	16,016	16,016
R-square	0.266	0.291	0.551	0.567

*, **, and *** indicate the 10%, 5%, and 1% statistical significance levels.

The values in brackets are robust standard errors after clustering at the household level.

About the characteristics of household head, the impact of age on rural household consumption relative deprivation shows a positive U-shaped trend, with consumption inequality being lower in middle age than in youth and old age, suggesting that the consumption level of middle-aged households is relatively high and that household consumption expenditures show heterogeneity over the life cycle. On average, consumption inequality is higher among male-headed households, a result that may be related to the generally lower willingness to consume among men. Married households have higher levels of household consumption expenditure because they are more risk-resistant and thus have less incentive to save. Educational attainment and income tend to be positively correlated, with higher education levels triggering higher levels of household consumption and thus lower levels of consumption inequality. The negative correlation between the household head’s employment status and the degree of consumption inequality suggests that employment may mitigate consumption inequality by augmenting household income. Individuals with better self-rated health status tend to exhibit lower levels of household consumption inequality, potentially due to the positive correlation between good health and higher productivity and earning potential. In terms of household characteristics, population size has a significantly negative impact on consumption inequality, confirming that household size is an important means for rural households to protect themselves against economic risks. Income, financial assets, and property have a significantly negative impact on consumption inequality, as household income and assets increase, the wealth effect increases while liquidity constraints and precautionary saving incentives diminish, leading households to increase consumption. Participation in pension insurance in rural areas, on the other hand, significantly increases consumption inequality, consistent with the findings of Li and Zang (34) that pension income in rural areas with relatively limited sources of income widens the gap in transfers between households and creates stronger perceptions of consumption deprivation among disadvantaged households that do not participate in pension insurance. The impact of household age structure on consumption inequality is multifaceted: a higher proportion of older adult individuals in the family leads to greater household consumption inequality, while a higher proportion of family members in the education phase results in lower household consumption inequality. The level of consumption inequality decreases as the family’s socioeconomic status increases. Control variables at the regional level indicate that residents in areas with higher per capita GDP, more per capita health expenditure, and in the eastern region experience relatively lower levels of consumption inequality.

### 4.2 Regression tests by income quartile

To examine the consumption response across different income strata following the implementation of urban–rural integrated health insurance, household consumption expenditure’s logarithm serves as the outcome variable. Regression analysis is conducted both on the entire sample and on five sub-samples categorized by income quartiles: low-income, lower-middle-income, middle-income, middle-upper-income, and high-income groups based on the 20th, 40th, 60th, and 80th quartiles of household income. This approach aims to ascertain whether the integration of urban and rural health insurance disproportionately stimulates consumption among higher-income households. Table 3 showcases the results, where column (1) indicates a significant increase in rural residents’ consumption expenditures attributable to urban–rural health insurance integration. Columns (2) to (6) further reveal the heterogeneous consumption effects across income levels: while health insurance coordination notably reduces consumption expenditures among low-income households, it significantly boosts consumption levels among middle-income, middle-upper-income, and high-income households. The magnitude of this effect escalates with higher household income quartiles, whereas the consumption expenditure of middle and low-income groups remains unaffected. This suggests that urban–rural health insurance integration primarily unlocks consumption potential among rural middle- and high-income groups, with limited incentives for consumption expenditure among low-income groups.

**Table 3:** Regression results by income quartile.

Variable	(1)	(2)	(3)	(4)	(5)	(6)
	Total	q0-q20	q20-q40	q40-q60	q60-q80	q80-q100
*insurance*	0.101** (0.043)	−0.095* (0.049)	−0.039 (0.043)	0.112*** (0.038)	0.173*** (0.036)	0.221*** (0.039)
controls	YES	YES	YES	YES	YES	YES
area FE	YES	YES	YES	YES	YES	YES
time FE	YES	YES	YES	YES	YES	YES
Obs	16,062	3,260	3,162	3,212	3,280	3,148
R-square	0.172	0.164	0.186	0.147	0.164	0.246

*, **, and *** indicate the 10%, 5%, and 1% statistical significance levels.

The values in brackets are robust standard errors after clustering at the household level.

### 4.3 Heterogeneity tests

The influence of urban–rural health insurance coordination on consumption inequality may encompass not only overall level effects but also heterogeneous structural effects across various consumption categories. Furthermore, its impact on the consumption gap among rural households may reveal nuanced age hierarchies. This section delves into the diverse effects of urban–rural health insurance coordination on rural consumption inequality.

#### 4.3.1 Structural effects of consumption inequality

Consumption expenditure can be classified as subsistence, development, and enjoyment consumption. We use the Kakwani index, which measures the relative inequality indices of households’ subsistence, development, and enjoyment consumption respectively, as an outcome variable to examine the effects of the urban–rural health insurance integration policy on relative deprivation in different types of consumption, and the regression results are shown in Table 4, columns (1) -column (3). It is easy to find that the urban–rural health insurance coordination policy has a significant expansion effect on both subsistence and enjoyment consumption inequality of rural households, and the comparison of the degree reveals that the expansion of subsistence consumption inequality is greater; on the contrary, development consumption inequality is not significantly affected by the coordination. The reasons for this may be: on the one hand, education expenditure is both the main item of development consumption in rural households and certainty for households with children in the education stage, and households generally invest less in development consumption other than that, thus the differences are limited. On the other hand, the consumption structure of rural households in China generally reflects a high proportion of subsistence consumption represented by food, and the implementation of urban–rural health insurance coordination in this context will have a greater impact on the release of consumption potential of the relatively high-income group, which has led to an intensification of the differentiation of subsistence consumption.

**Table 4:** Heterogeneity tests results.

Variable	(1)	(2)	(3)	(4)	(5)	(6)	(7)
	RDLIV	RDDEV	RDENJ	under 30	31–45	46–60	over 61
*insurance*	0.037*** (0.006)	−0.003 (0.007)	0.024*** (0.007)	0.018 (0.022)	0.016 (0.011)	0.033*** (0.009)	0.058*** (0.011)
controls	YES	YES	YES	YES	YES	YES	YES
area FE	YES	YES	YES	YES	YES	YES	YES
time FE	YES	YES	YES	YES	YES	YES	YES
Obs	16,016	16,016	16,016	1,676	4,956	6,824	4,208
R-square	0.330	0.311	0.288	0.275	0.257	0.303	0.407

*, **, and *** indicate the 10%, 5%, and 1% statistical significance levels.

The values in brackets are robust standard errors after clustering at the household level.

#### 4.3.2 Life-cycle effect of consumption inequality

The impact of urban–rural health insurance integration on consumption inequality across different age cohorts is examined in Table 4, columns (4)–(7). The analysis categorizes individuals into four age brackets: under 30 years old, 31–45 years old, 46–60 years old, and over 61 years old. Findings reveal a significant widening of consumption relative deprivation among individuals aged 46–60 and those aged 61 or older due to urban–rural health insurance coordination. Particularly pronounced is the consumption deprivation observed among households in the 61 or older age group, underscoring the considerable expansion of consumption inequality attributable to the implementation of health insurance coordination. This exacerbation of consumption inequality in the 46–60 or older age groups suggests a heightened sensitivity to policy changes among older individuals, likely influenced by their elevated health risks. Conversely, younger age groups exhibit comparatively lower sensitivity to alterations in health insurance policies owing to their reduced health risks.

### 4.4 Robustness tests

#### 4.4.1 Parallel trend test

The premise of using a staggered DID model lies in the consistent trend of change between the treatment group and the control group before the implementation of the policy. If there is no discernible disparity in consumption inequality trends between the treatment and control groups before the enactment of the urban–rural health insurance integration policy, yet a significant discrepancy emerges post-implementation, it suggests that the policy itself influences consumption inequality. Given variations in the timing of policy implementation across regions, this study employs the event study method to conduct a multi-period DID analysis. This involves introducing a dummy variable spanning from four periods before to two periods after policy implementation, alongside the interaction term of the urban–rural health insurance integration policy, to regress on consumption inequality. The model is formulated as follows:


(4) $$RD_{ijt}=\alpha +\overset{2}{\underset{k=-4}{\sum }}\beta_{k}\mathrm{trea}t_{ij}\times post_{k}+\beta_{3}X_{ijt}+\lambda_{j}+u_{t}+\lambda_{j}\times u_{t}+\varepsilon_{ijt}$$

**Figure 2:**
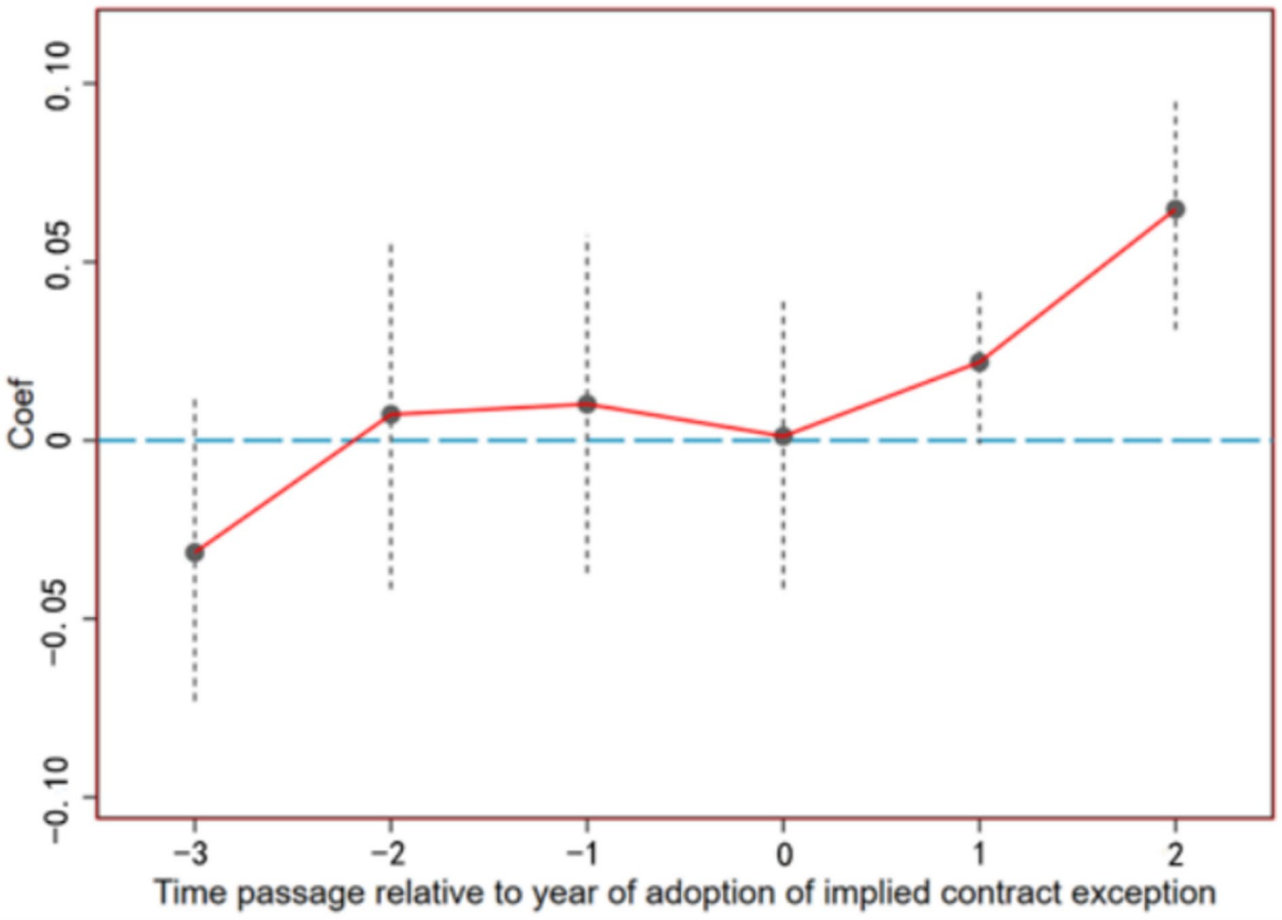
Parallel trend test.

In Equation 4, *treat_ij_* denotes whether household *i* located in district *j* has implemented urban–rural health insurance coordination during the survey period, with a value of 1 assigned to implementation and 0 otherwise. The variable *post_k_* represents the year dummy variable, ranging from −4 to 2, indicating 1–4 periods before policy implementation, the implementation year, and 1–2 period post-implementation, respectively. Other parameters are defined analogously to Equation 3. Parameter *β_k_* reflects the impact of insurance integration on consumption disparities between coordinated and non-coordinated regions, with the 4th year pre-implementation serving as the base period. As depicted in Figure 2, prior to integration, the 95% confidence intervals of the estimated coefficients*β_−3_*, *β_−2_,* and *β_−1_* do not significantly deviate from 0, indicating that there is no significant difference in consumption inequality between the treatment and control groups, thus satisfying the parallel trends assumption. In terms of the dynamic effects of the policy, in the year of policy implementation, the effect of urban–rural health insurance coordination on consumption inequality has not yet stabilized, as evidenced by the 95% confidence interval of *β_0_* not significantly deviating from 0. However, 1–2 period after the policy was rolled out, the impact coefficients of urban–rural health insurance coordination significantly increased and remained positive, suggesting that the policy has the effect of expanding rural consumption inequality and has a certain degree of lag, as indicated in the benchmark regression.

#### 4.4.2 Placebo test

**Figure 3:**
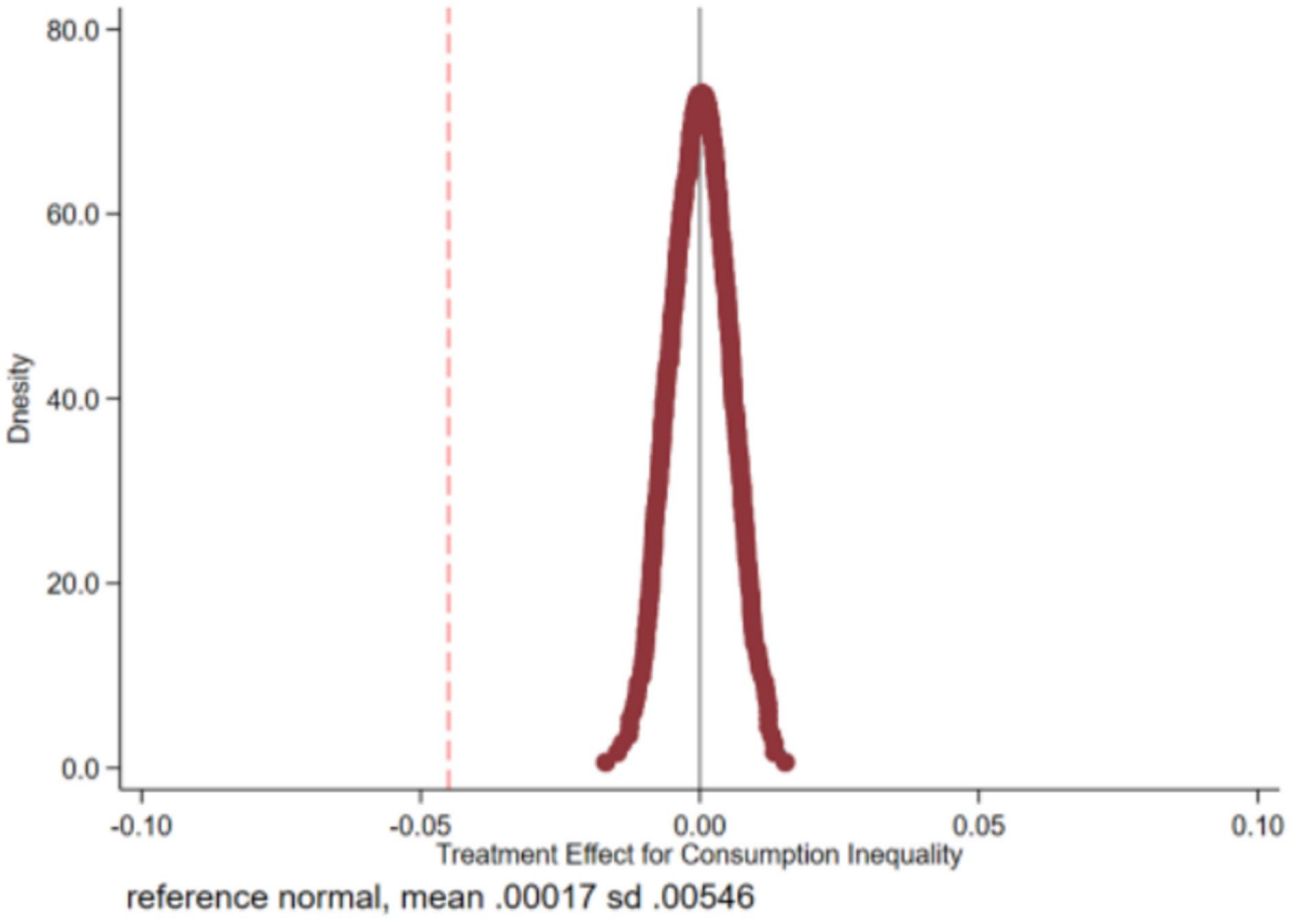
Non-parametric permutation test.

In this study, we utilize a non-parametric permutation test to mitigate the influence of other policy shocks. Additionally, we conduct a placebo test by randomly assigning households in the sample to either implement or not implement the urban–rural health insurance coordination policy. The sample comprises 1,388 households enrolled in the urban–rural health insurance scheme. Among these, 1,388 households are randomly designated as the false treatment group, while the remaining households constitute the false control group. This process is repeated 500 times to generate 500 randomized treatment and control groups, which are subsequently re-estimated using the generated randomized samples following Equation 3. Given that the false treatment group is not grounded in actual implementation, health insurance integration theoretically exerts no significant impact on consumption inequality (*β_1_^false^* = 0). Conversely, a statistically significant deviation of *β_1_^false^* from 0 indicates potential identification bias in the baseline model. Test results illustrated in Figure 3 demonstrate that the mean values of the estimated coefficients derived from the 500 regressions predominantly cluster around 0. Conversely, the estimated coefficients from the benchmark regression (depicted by the red dotted line) distinctly deviate from this pattern, underscoring the significant causal effect of the urban–rural health insurance integration policy on rural consumption relative deprivation.

#### 4.4.3 PSM-DID test

Apart from a few pioneering pilot cities, the implementation of the integrated urban and rural health insurance policy and the specific timing of its execution are uniformly determined by the central government. This policy framework constitutes a “quasi-natural experiment” for household decision-making. Such a policy implementation structure significantly mitigates endogeneity issues, as it resembles the conditions of a randomized experiment. The DID model is leveraged to exploit the plausibly exogenous variation in policy implementation across different regions and time periods, thereby providing a cleaner identification strategy for evaluating policy effects. The Propensity Score Matching (PSM) approach identifies samples from the control group that are similar to the treatment group in terms of observable characteristics, constructing a counterfactual scenario. This method helps to correct for selection bias due to observable variables, enhancing the external validity of the research findings. By combining PSM with the DID method, this study aims to further reduce the impact of sample selection bias and more effectively address potential endogeneity issues. Initially, we used individual, household, and regional characteristics for propensity score matching, employing 1:1 nearest neighbor, kernel, and radius matching techniques to create comparable treatment and control groups. Following this, we applied staggered DID estimation to the matched samples to assess the policy’s impact. The results presented in columns (1) to (3) of Table 5 emphasize that the standard errors of the PSM-DID estimates are reduced compared to those of the basic DID estimates. Furthermore, the regression coefficients of the interaction term DID in the PSM-DID estimates are significantly larger than those obtained from the basic DID estimates. In general, the PSM-DID estimates are of higher quality and more reliable, having passed the robustness test. The significant positive impact of implementing an integrated urban–rural health insurance policy in rural areas on consumption inequality is observed regardless of the matching method used, whether it be 1:1 nearest neighbor matching, kernel matching, or radius matching.

**Table 5:** Robustness tests.

Variable	PSM-DID test			Controlling for time-based differences			Tobit-DID model	Substitute outcome variable	Household FE	CSDID
	Nearest neighbor matching	Kernel matching	Radius matching	Only household-level time trends	Only city-level time trends	both household and city-level time trends				
	(1)	(2)	(3)	(4)	(5)	(6)	(7)	(8)	(9)	(10)
*insurance*	(0.011)	0.042*** (0.011)	0.040*** (0.011)	0.038*** (0.011)	0.037*** (0.012)	0.038***	0.067*** (0.011)	0.055** (0.023)	0.032** (0.016)	0.040** (0.019)
controls	YES	YES	YES	YES	YES	YES	YES	YES	YES	YES
area FE	YES	YES	YES	YES	YES	YES	YES	YES	YES	YES
time FE	YES	YES	YES	YES	YES	YES	YES	YES	YES	YES
Obs	11,104	11,104	11,104	16,016	16,016	16,016	16,016	16,016	16,016	16,016
R-square	0.312	0.291	0.324	0.353	0.353	0.353	0.236	0.235	0.235	0.278

*, **, and *** indicate the 10%, 5%, and 1% statistical significance levels.

The values in brackets are robust standard errors after clustering at the household level.

#### 4.4.4 Controlling for time-based differences between groups

To further test for endogeneity, this paper first introduces interaction terms between initial household differences such as head gender, family income, financial assets, and population size with a linear time trend to control for the unobservable factors that may vary year by year between the treatment and control groups at the household level. The results, as shown in column (4) of Table 5, indicate that the coefficient for the urban–rural health insurance integration remains significantly positive. Secondly, an interaction term between the city-level dummy variable and time is introduced to address the potential time-varying impact of city characteristics on household consumption. As shown in column (5), the coefficient for the urban–rural integrated health insurance policy is also significantly positive, confirming the policy’s expansionary effect on consumption inequality. Lastly, column (6) includes interaction terms between household and regional-level variables with a linear time trend. The results indicate that after controlling for the time trends of the original differences between the treatment and control groups, the expansionary effect of the urban–rural health insurance integration on consumption inequality remains statistically significant.

#### 4.4.5 Substitution method, dependent variable or fixed effect

Firstly, given that the outcome variable ranges between 0 and 1, this study employs the staggered DID method based on the restricted outcome variable (Tobit) model to reexamine the relationship between the implementation of urban–rural health insurance integration policy and consumption inequality in rural areas. Column (7) of Table 5 reveals a significantly positive coefficient. Secondly, refer to Deng and Yang (6), the Kakwani relative deprivation index is transformed into five ordered levels of consumption deprivation by dividing the index into intervals of 0.2. This transformed index is then re-evaluated as an outcome variable utilizing the Ordered Probit model. Column (8) of Table 5 demonstrates a significantly positive coefficient, consistent with the findings of the baseline regression analysis. Moreover, by shifting the individual fixed effects from the city level to the household level and employing household fixed effects, time fixed effects, and their interaction terms in the baseline regression of Equation 3, the findings indicate that despite the increase in standard errors, the urban–rural medical insurance integration still significantly amplifies consumption inequality at the 5% significance level (Column 9).

#### 4.4.6 Robustness tests for heterogeneity of treatment effects

Current literature suggests that variations in the timing and manner of treatment acceptance among the treated group can lead to heterogeneity in treatment effects over time, challenging the robustness of staggered DID estimates (56). Specifically, differences in the treated timing among the treatment group may cause those treated earlier to serve as a control group for those treated later, leading to biased policy effects. Considering the variations in the timing and manner of accepting integrated urban–rural health insurance among different households, individual characteristics, and environmental factors may lead to diverse manifestations of treatment effects. This study adopts the estimation method proposed by Callaway and Sant’Anna (57), dividing the sample into different subgroups and estimating the average treatment effect for each subgroup. The average treatment effects from different groups are then combined according to a specific aggregation strategy. This strategy primarily involves reducing the weight of the average treatment effect for groups that may have estimation biases, excluding the aforementioned unreasonable control relationships, and estimating the dynamic characteristics of treatment effects. The estimation results using the CSDID model, as shown in column (10) of Table 5, indicate that even after controlling for the heterogeneity of treatment effects, the impact of health insurance integration on household consumption inequality remains significantly positive, with a coefficient close to the baseline regression results.

### 4.5 Spatial spillover effects tests

In this section, we explore whether the urban–rural health insurance integration policy has generated spillover effects on neighboring areas. On the one hand, the implementation of the policy may lead to a concentration of medical resources in pilot areas, thereby attracting residents from surrounding non-pilot areas to seek medical treatment and increasing the medical burden on these areas. On the other hand, it may also be due to policy spillover effects, where medical institutions in neighboring areas follow the reform measures of the pilot areas, improving service quality or reducing costs, or due to sharing a broader medical service market, the reform effects of the pilot areas have spread to neighboring cities. To test whether the urban–rural health insurance integration policy has affected neighboring areas, we evaluate the policy’s impact on five new treatment groups outside the pilot cities: (0–150) km, (150–200) km, (200–250) km, (250–300) km, and (300–350) km. These five new treatment groups include a varying number of non-pilot cities.

As we found in Figure 1 that the impact of the urban–rural health insurance integration policy has a lag, we present in Table 6 two time points after the policy implementation, namely the year the policy was launched (Panel A) and two periods after the policy implementation (Panel B).

**Table 6:** Spillover effects regressions results.

Variable	(1)	(2)	(3)	(4)
Panel A				
*insurance*	0.039*** (0.011)	0.036*** (0.012)	0.033*** (0.012)	0.023*** (0.008)
*nearby (0-150) km*	0.013 (0.021)	0.015 (0.021)	0.021 (0.022)	0.018* (0.023)
*nearby (150-200) km*		0.022 (0.019)	0.028 (0.020)	0.015 (0.011)
*nearby (200-250) km*			0.013 (0.020)	0.020 (0.021)
*nearby (250-300) km*				0.001 (0.017)
R-square	0.466	0.461	0.477	0.477
Panel B				
*insurance*	0.047*** (0.013)	0.045*** (0.014)	0.039*** (0.014)	0.038** (0.015)
*nearby (0-150) km*	0.022*** (0.007)	0.029*** (0.007)	0.025*** (0.008)	0.028*** (0.011)
*nearby (150-200) km*		0.023** (0.011)	0.020*** (0.007)	0.012*** (0.004)
*nearby (200-250) km*			0.013*** (0.005)	0.006*** (0.002)
*nearby (250-300) km*				0.005 (0.003)
controls	YES	YES	YES	YES
area FE	YES	YES	YES	YES
time FE	YES	YES	YES	YES
Obs	11,104	11,104	11,104	11,104
R-square	0.461	0.491	0.429	0.453

*, **, and *** indicate the 10%, 5%, and 1% statistical significance levels.

The values in brackets are robust standard errors after clustering at the household level.

As shown in Table 6, we find that the urban–rural health insurance integration policy not only widens consumption inequality in pilot areas but also has adverse spillover effects on neighboring non-pilot cities within 300 km of the pilot cities. Like the direct effect on pilot cities (Figure 2), the spillover effects also show a lag. In Panel A, we assume that the policy effect starts from the year the policy was launched and estimate the impact on the pilot and neighboring cities. The results in Column 4 of Panel A show that there is no significant widening effect in areas more than 150 km away from the pilot cities. In contrast, in Panel B, when considering the impact two periods after the policy implementation, we find strong evidence of spillover effects, that is, the spillover effects significantly widen within 250 km of the pilot cities. This again indicates that the widening impact of the urban–rural health insurance integration policy on consumption inequality becomes more apparent after a period of policy implementation. Using the results from Column 4 of Panel B, we find that as the distance from the pilot area increases, the spillover effect gradually decreases, thus the spillover radius appears to be more than 0–250 km. The above results suggest that neighboring areas are affected by the urban–rural health insurance integration policy because they share a medical service market, which means that the increase in consumption inequality in pilot cities also affects neighboring cities. This implies that the estimates of consumption inequality increase in pilot cities reported in the benchmark regression may be biased downwards in absolute terms because neighboring cities in the control group are also affected by the policy effect.

## 5 Mechanism analysis

This study delves into the potential pathways by which urban–rural health insurance coordination influences consumption inequality. It examines the impact on health service utilization and healthcare burdens, precautionary savings, and variations in health insurance funding modalities.

### 5.1 Healthcare service utilization

This section investigates the impact of urban–rural health insurance integration on healthcare motivation and service utilization across various income strata. Drawing insights from Chang et al. (50), who utilize outpatient visit probability and hospitalization probability to gage healthcare motivation, as well as the logarithm of medical out-of-pocket expenses and the ratio of medical out-of-pocket expenses to household non-medical consumption to measure healthcare service utilization, regression analysis is conducted by introducing the interaction term of urban–rural health insurance integration with logarithmic household income. Results presented in Table 7 reveal noteworthy patterns. In columns (1) and (2) analyzing healthcare motivation, the implementation of health insurance coordination significantly boosts the likelihood of household consultations and hospitalizations. However, this enhancement effect diminishes significantly with rising income levels, suggesting that while urban–rural health insurance coordination bolsters healthcare-seeking among rural households, its impact is more pronounced for low-income groups. From the analysis of health service utilization in columns (3) and (4), it can be seen that after the implementation of integration, out-of-pocket expenses and the ratio of medical out-of-pocket expenses to household non-medical consumption both increased at the 1% test level. The proportion of out-of-pocket expenses also showed a significant decrease with increasing income, indicating that the integration correspondingly increased the medical burden on low-income groups. This phenomenon can be attributed to the fact that low-income individuals, constrained by financial limitations, often adopt a medical avoidance mindset. The integration of health insurance substantially increases healthcare accessibility for these groups, thereby unleashing pent-up medical needs previously suppressed by budget constraints. Consequently, there is a surge in medical out-of-pocket expenses, leading to a crowding-out effect on current household consumption. Conversely, the rise in out-of-pocket medical expenses for high-income households remains more moderate, indicating that increased medical service utilization among this cohort has minimal crowding-out effects on household consumption.

**Table 7:** Impact of the urban–rural health insurance integration policy on health service utilization.

Variable	(1)	(2)	(3)	(4)
	Healthcare motivation		Health service utilization	
	Outpatient	Hospitalization	OOP expenses	Ratio of OOP
*insurance*	0.118** (0.052)	0.115** (0.055)	0.109*** (0.027)	0.146*** (0.031)
*insurance × lnincome*	−0.008** (0.004)	−0.007** (0.004)	−0.013 (0.009)	−0.011*** (0.003)
controls	YES	YES	YES	YES
area FE	YES	YES	YES	YES
time FE	YES	YES	YES	YES
Obs	15,033	14,509	13,407	13,407
R-square	0.333	0.324	0.335	0.316

*, **, and *** indicate the 10%, 5%, and 1% statistical significance levels.

The values in brackets are robust standard errors after clustering at the household level.

Table 8 presents the dynamic effects of policy implementation on healthcare motivations and service utilization. In terms of healthcare incentives, columns (1) and (2) indicate that before the implementation of the coordinated health insurance scheme, the probability of outpatient and hospitalization is significantly higher for high-income households compared to low-income households during periods 1–2 and 2–3. However, no statistically significant differences exist in consultation and hospitalization probabilities between income groups during the implementation period of the coordinated scheme. Furthermore, household consultation and hospitalization probabilities decrease with rising income during the first year of policy implementation, with policy effects no longer significant after the second period of coordination. This suggests that the short-term expansionary impact of urban–rural health insurance integration on healthcare accessibility for low-income households is notably pronounced. Regarding healthcare service utilization, columns (3) and (4) demonstrate that before healthcare insurance integration, both out-of-pocket expenses and the proportion of out-of-pocket expenses increased significantly with household income, with healthcare consumption notably higher among high-income groups. However, in the year of policy implementation and beyond, out-of-pocket expenses no longer exhibit significant statistical differences across income groups, and the proportion of out-of-pocket expenses gradually decreases with income. This indicates that the urban–rural health insurance integration policy has mitigated disparities in absolute out-of-pocket medical expenses between income groups. Nevertheless, the burden of out-of-pocket medical expenses on low-income households remains heavier than on high-income households, exacerbating inequality by squeezing disposable income among low-income groups and suppressing consumption expenditures to a greater extent.

**Table 8:** Dynamic impact of urban–rural health insurance integration policy on health service utilization.

Variable	(1)	(2)	(3)	(4)
	Healthcare motivation		Health service utilization	
	Outpatient	Hospitalization	OOP expenses	Ratio of OOP
*post-4 × lnincome*	0.000 (0.001)	−0.001 (0.001)	0.008*** (0.001)	0.003*** (0.001)
*post-3 × lnincome*	0.001 (0.001)	0.003*** (0.001)	0.005** (0.002)	0.003*** (0.001)
*post-2 × lnincome*	0.003*** (0.001)	0.002** (0.001)	0.011* (0.006)	0.003*** (0.001)
*post-1 × lnincome*	0.002** (0.001)	0.000 (0.001)	0.015** (0.006)	0.004*** (0.001)
*post0 × lnincome*	−0.007 (0.005)	0.001 (0.004)	−0.017 (0.035)	−0.013** (0.006)
*post1 × lnincome*	−0.013*** (0.005)	−0.003*** (0.001)	−0.033 (0.037)	−0.016*** (0.006)
*post2 × lnincome*	−0.007 (0.006)	0.001 (0.005)	−0.017 (0.045)	−0.015* (0.008)
*post3 × lnincome*	−0.002 (0.006)	0.007 (0.005)	0.002 (0.045)	−0.017* (0.009)
controls	YES	YES	YES	YES
area FE	YES	YES	YES	YES
time FE	YES	YES	YES	YES
Obs	15,033	14,509	13,090	13,038
R-square	0.110	0.112	0.219	0.212

*, **, and *** indicate the 10%, 5%, and 1% statistical significance levels.

The values in brackets are robust standard errors after clustering at the household level.

### 5.2 Precautionary savings

Households with higher initial endowments tend to engage in more precautionary savings (52). Does urban–rural coordinated health insurance have a more pronounced effect on releasing precautionary savings in the high-consumption group compared to the low- and middle-consumption groups? Sample grouping regressions are conducted with precautionary savings as the outcome variable, categorized by consumption level quintiles. Precautionary saving is proxied by the proportion of liquid assets held by households. The regression outcomes in Table 9 reveal that for consumption level quintiles 0–20 and 20–40, the regression coefficients of urban–rural coordinated health insurance on precautionary savings are statistically insignificant. However, for quintiles 40–60, 60–80, and 80–100, the policy significantly reduces household precautionary savings, with this releasing effect expanding as consumption levels increase. This underscores the higher prevalence of precautionary savings among middle- and high-consumption groups in rural areas, and the subsequent release of savings motivation following the implementation of health insurance integration due to enhanced health insurance benefits, thereby promoting consumption. Conversely, it is challenging to diminish the incentive for precautionary savings among low-income groups, and the integration of the health insurance scheme has yet to unlock their consumption potential.

**Table 9:** Impact of the urban–rural health insurance integration policy on precautionary savings.

Variable	(1)	(2)	(3)	(4)	(5)
	Precautionary savings				
	q0-q20	q20-q40	q40-q60	q60-q80	q80-q100
*insurance*	−0.001 (0.004)	−0.005 (0.005)	−0.010** (0.005)	−0.012** (0.005)	−0.015*** (0.005)
controls	YES	YES	YES	YES	YES
area FE	YES	YES	YES	YES	YES
time E	YES	YES	YES	YES	YES
Obs	3,991	3,039	3,062	3,154	3,189
R-square	0.990	0.979	0.972	0.956	0.961

*, **, and *** indicate the 10%, 5%, and 1% statistical significance levels.

The values in brackets are robust standard errors after clustering at the household level.

### 5.3 Funding modalities analysis

Two primary funding modalities exist for urban–rural integrated health insurance: the single-tier system and the multi-tier system. This section examines whether these different financing modes influence the degree of consumption inequality. The sample is divided into single-tier and multi-tier regions, and regression analysis is conducted according to Equation 3. The results presented in Table 10 reveal that urban–rural health insurance integration has a significantly higher impact on consumption inequality in single-tier regions compared to multi-tier regions, suggesting that the single-tier model exacerbates consumption inequality to a greater extent in rural areas. A plausible explanation is that, amidst low disposable income, the multi-tiered model accounts for income disparities among rural households by offering flexible contribution standards, enabling low-income households to choose affordable participation options based on their financial circumstances. This approach alleviates budget constraints, prevents premium increases from crowding out non-medical consumption, and empowers individuals with the autonomy to make independent choices. Consequently, this contributes to reducing consumption inequality between urban and rural areas. Moreover, individual autonomy in selecting contribution standards helps mitigate moral hazards and excessive medical demands resulting from passive payment of high premiums, fostering moderate consumption growth while maintaining stability. This underscores the effectiveness of the multi-tiered system as a transitional model for healthcare integration within the urban–rural healthcare framework, tailored to local needs.

**Table 10:** Regression results distinguishing between funding modalities.

Variable	(1)	(2)
	Single-tier	Multi-tier
*insurance*	0.041*** (0.007)	0.021* (0.012)
controls	YES	YES
area FE	YES	YES
time FE	YES	YES
Obs	12,842	2,108
R-square	0.318	0.363

*, **, and *** indicate the 10%, 5%, and 1% statistical significance levels.

The values in brackets are robust standard errors after clustering at the household level.

## 6 Discussion

This study provides empirical evidence on the complex dynamics of China’s urban–rural health insurance integration policy, particularly its effects on healthcare access, consumption patterns, and economic inequality. The integration of urban and rural health insurance has significantly increased healthcare utilization among low-income populations. This finding aligns with studies on similar health insurance reforms, such as the Affordable Care Act (ACA) in the United States, which showed a substantial increase in healthcare utilization among low- and middle-income groups (29, 37). Theoretically, expanding health insurance coverage should reduce economic barriers to healthcare access, particularly for vulnerable groups, thereby improving overall social welfare.

However, this study also reveals a counterintuitive effect: the policy appears to exacerbate consumption inequality, particularly among low-income households. This paradox highlights the limitations of health reforms that rely solely on expanding coverage without considering their broader economic impacts. First, increased healthcare utilization generates indirect costs. Low- and middle-income groups may face economic pressures due to higher medical expenses, which could suppress the potential for consumption growth. Second, the integration of health insurance primarily benefits households with medium to high consumption levels, rather than impoverished groups, indicating that the policy may inadvertently widen the gap between income groups. Finally, the unified payment and reimbursement model has absorbed economic resources that could otherwise have been used to improve consumption for low-income households. These findings are consistent with the “reverse subsidy” mechanism highlighted in the literature, where high-income groups receive disproportionately greater benefits from public policies compared to low-income groups—a trend widely observed in global health insurance integration systems (9, 10, 50).

From a theoretical perspective, this study contributes to understanding how health insurance reform affects not only health outcomes but also broader economic behaviors, particularly consumption patterns. It emphasizes the need for a more nuanced approach that not only focuses on healthcare accessibility but also takes into account household economic welfare and the broader socioeconomic context.

The findings of this study provide important implications for policymakers and health insurance providers. First, while the integration of urban and rural health insurance policies has improved healthcare access, it has also unintentionally exacerbated consumption inequality. Policymakers must, therefore, adopt a more equitable approach when designing health insurance systems to alleviate the economic burdens on low-income families. This could be achieved through the implementation of a tiered health insurance payment structure that considers household income, health status, and age, thereby reducing economic pressure on vulnerable groups. Furthermore, the government could provide subsidies to low-income households, particularly those in rural areas, to help offset increased medical expenditures and protect household consumption capacity.

In addition, the study highlights the necessity of more targeted healthcare assistance policies. Increasing reimbursement rates for critical illness insurance and expanding coverage for high-risk groups, including the older adult and individuals with chronic conditions, can effectively alleviate the financial pressure on these groups. The study also emphasizes that addressing consumption inequality requires more than just improving healthcare access—it necessitates the introduction of complementary policies in rural areas to enhance overall consumption capacity. Initiatives such as improving the quality of rural healthcare services, promoting the adoption of commercial health insurance, and leveraging technological innovations to optimize healthcare delivery can further enhance the equity of health insurance reforms.

Finally, the spillover effects observed in non-pilot cities suggest that health insurance integration in pilot regions may have unintended consequences for neighboring areas. The direct and indirect impacts of the policy may manifest in neighboring cities after a certain lag, highlighting the need for more comprehensive and region-specific strategies during policy implementation to avoid exacerbating imbalances in healthcare and economic outcomes across regions.

Despite providing valuable insights, this study has several limitations. First, although the study focuses on rural populations in China, the findings may not be directly applicable to other countries, especially those with different health insurance systems, income distributions, or healthcare structures. However, as the most populous and economically diverse developing country, China’s experience with health insurance integration provides valuable lessons for other developing nations, particularly those seeking to expand coverage within limited financial resources. Second, the study primarily focuses on consumption and economic behavior, without fully incorporating other health-related dimensions, such as mental health, healthcare quality, and long-term health outcomes. Incorporating these health dimensions in future evaluations of health insurance integration would provide a deeper understanding of the trade-offs involved in policy design. Lastly, the study does not adequately explore the dynamic interactions between health insurance reform and other policy areas, such as pension systems, labor markets, and social assistance policies, which may further affect welfare distribution among different groups. Future research could extend the time and geographic scope of the data, include additional welfare indicators, and explore the interactions between health insurance policies and other social policies to further refine the theoretical and empirical understanding of this field. Moreover, cross-national and cross-regional comparative studies could help identify best practices and refine theoretical models of health insurance reform, particularly in the context of emerging economies.

## 7 Conclusion

This study systematically explores the impact of the urban–rural health insurance integration policy on consumption inequality among rural households and its underlying mechanisms using data from the China Family Panel Studies from 2012 to 2018, employing a staggered difference-in-differences model. To our knowledge, this is one of the few studies that examine the association between a national health insurance expansion and households’ perceived fairness based on a consumption inequality perspective. The findings indicate a significant increase in household consumption relative deprivation due to the policy while ensuring equitable access to enhanced health insurance benefits for rural households. Health insurance coordination not only elevates consumption levels among middle- and high-income groups but also reduces consumption expenditures for low-income groups. The conclusions drawn from this paper reveal that in designing and adjusting health insurance policies, policymakers should take into account the affordability and consumption capacity of low-income groups to ensure the fairness and effectiveness of the policies. In addition, the implementation of policies requires attention to its far-reaching impact on the consumption and economic well-being of households. Future research should place greater emphasis on the construction of a systematic health insurance evaluation index system, the similarities and differences in health insurance system reforms across different countries and regions, as well as the flexible adaptation of health insurance systems in the face of demographic aging and the emergence of new business models.

## Data Availability

Publicly available datasets were analyzed in this study. These data can be found in the China Family Panel Study (CFPS) database maintained by the China Social Science Survey Center of Peking University, accessible at: https://cfpsdata.pku.edu.cn/#/home.
